# Diversity and Biomineralization Potential of the Epilithic Bacterial Communities Inhabiting the Oldest Public Stone Monument of Cluj-Napoca (Transylvania, Romania)

**DOI:** 10.3389/fmicb.2017.00372

**Published:** 2017-03-07

**Authors:** Adrian-Ştefan Andrei, Manuela R. Păuşan, Tudor Tămaş, Nicolae Har, Lucian Barbu-Tudoran, Nicolae Leopold, Horia L. Banciu

**Affiliations:** ^1^Department of Molecular Biology and Biotechnology, Babeş-Bolyai UniversityCluj-Napoca, Romania; ^2^Institute of Hydrobiology, Department of Aquatic Microbial Ecology, Biology Center of the Academy of Sciences of the Czech RepublicČeské Budějovice, Czechia; ^3^Department for Internal Medicine, Medical University of GrazGraz, Austria; ^4^Department of Geology, Babeş-Bolyai UniversityCluj-Napoca, Romania; ^5^Electron Microscopy Center, Babeş-Bolyai UniversityCluj-Napoca, Romania; ^6^Faculty of Physics, Babeş-Bolyai UniversityCluj-Napoca, Romania; ^7^Center for Systems Biology, Biodiversity, and Bioresources, Babeş-Bolyai UniversityCluj-Napoca, Romania

**Keywords:** limestone statue, epilithic microbiota, bacterial cultivation, carbonatogenesis, vaterite, calcite

## Abstract

In this study, we investigated the biomineralization potential and diversity of the epilithic bacterial communities dwelling on the limestone statue of Saint Donatus, the oldest public monument of Cluj-Napoca city (Transylvania region, NW Romania). Their spatial distribution together with phylogenetic and metabolic diversity, as well as their capacity to precipitate calcium carbonate was evaluated by combining molecular and phenotypic fingerprinting methods with X-ray diffraction, Fourier transform infrared spectroscopy, and scanning electron-microscopy analyses. The results of real-time quantitative PCR, molecular fingerprinting and community-level physiological profiling showed that diverse and abundant bacterial assemblages that differ in relation to their collection site colonized the statue. The cultivation and molecular identification procedures allowed the characterization of 79 bacterial isolates belonging to Proteobacteria (73.4%), Firmicutes (19%), and Actinobacteria (7.6%). Amongst them, the 22 strains identified as being capable of calcium carbonate precipitation were found to belong mostly to *Bacillus* and *Pseudomonas* genera. We found that bacteria acted as nucleation sites, inducing the formation of nanoscale aggregates that were shown to be principally composed of vaterite. Furthermore, we expanded the current knowledge on culturable diversity of carbonatogenic bacteria by providing evidence for biogenic vaterite/calcite formation mediated by: *Pseudomonas synxantha*, *P. graminis*, *Brevibacterium iodinum*, *Streptomyces albidoflavus*, and *Stenotrophomonas chelatiphaga*. Overall, this study highlights the need to evaluate the carbonatogenetic potential of all the bacterial communities present on stone artwork prior to designing an efficient conservation treatment based on biomineralization.

## Introduction

Stone artworks of public (statues, sculptures, memorials) and private (gravestones, mausoleums) interest are built to last for extended periods of time. However, abiotic (humidity, thermal effects, salts and other chemicals, mechanical impact of winds and urban noises, light exposure, etc.) and biotic factors (biocolonization by macro- and microorganisms consortia) impassibly affect their structure and composition, leading to deterioration and esthetic alteration (Warscheid and Braams, 2000; Lisci et al., 2003; Steiger et al., 2014; Gil et al., 2015). Although limestone is one of the most preferred ornamental stones, it is especially prone to degradation and weathering due to its solubility and porous texture that confers susceptibility to corrosion and biodeterioration (Pentecost and Whitton, 2000; McNamara et al., 2006; Miller et al., 2012). Due to the fact that abiotically induced rock fissures within limestone are ideal traps for water and aerosols of mineral and organic origin, they favor microbial colonization by providing suitable habitats. Over time, the limestone monuments directly exposed to environment will develop lithobiontic trophic webs based on primary producers (i.e., photo- or chemolithoautotrophs) that will provide organic carbon sources for heterotrophic microorganisms (mainly bacteria and fungi) (Saiz-Jimenez, 1997; Gorbushina, 2007; Scheerer et al., 2009; Banciu, 2013). These lithobiontic consortia will act toward the expansion of internal fissures and by organic acid production (e.g., gluconic acid, lactic acid, etc.) will alter the chemical composition of the stone and lead to its decay (Banciu, 2013).

Microbial carbonate precipitation is a ubiquitous naturally occurring phenomenon, responsible for the occasional whiting events (Sondi and Juraěić, 2010), soil/sediment cementation and stromatolite genesis (Zhu and Dittrich, 2016). This process is mediated by diverse microorganisms through altering microenvironment chemistry (via various metabolic pathways such as ureolysis, ammonification, denitrification, sulfate reduction, etc.) or acting as highly effective nucleation templates. Inspired by its natural capability to cement (sands, soils, and minerals) and improve the mechanical properties of porous materials, microbial carbonate precipitation is envisioned as a promising biotechnology in stone monument restoration (Zhu and Dittrich, 2016). To date, two strategies involving bacteria capable of inducing CaCO_3_ precipitation have been used: one in which an axenic bacterial culture is applied directly on the stone, and another which utilizes the carbonatogenic potential of the resident microbial communities. The latter proved to be more reliable, since the usage of microorganisms that are not part of the existing microbial community may negatively interact with the existent microbial consortium (Ettenauer et al., 2011; Jroundi et al., 2012; Jagadeesha Kumar et al., 2013). Due to the fact that carbonate precipitation is highly influenced by environmental chemistry and bacterial metabolic activity, the spatial heterogeneity of microbial diversity may directly impact the mechanical and esthetic properties of the monument. Furthermore, the discrimination between different CaCO_3_ polymorphs may provide cues regarding their formation and consolidation strength (Daskalakis et al., 2015). As *Bacteria* represent the first and most diverse group of microorganisms that colonize ornamental stone, understanding their communities’ spatial distribution, structure and composition may lead to the development of more efficient conservation and restoration strategies (Cameotra and Dakal, 2012). Furthermore, the isolation and characterization of microbial strains dwelling on limestone monuments may provide the necessary means for envisioning environmentally friendly preservation methods based on biomineralization (i.e., carbonate bioprecipitation) (Jroundi et al., 2012; Dhami et al., 2014b; López-Moreno et al., 2014).

Deterioration of stoneworks belonging to the Romanian cultural heritage is scarcely explored, with most of the studies being focused on abiotic weathering of building stone from sepulchral (Koch et al., 2008; Brânzilă and Ştefan, 2009) or civil sites (Balog et al., 2014). In the present paper, we studied the lithobiontic bacterial communities dwelling on the baroque statue of Saint Donatus (Cluj, Romania), the first public monument of *Cluj-Napoca* city (Sabãu, 2002).

Due to the paucity of data on the usage of biomineralization in the restoration of Romanian public stone monuments and to the advanced state of deterioration of the Saint Donatus statue, we aimed to: (1) investigate whether the statue is colonized by one or multiple epilithic microbial communities (by using community metabolic and molecular fingerprinting) in order to (2) assess the potential usage of bioconsolidation (by evaluating the carbonatogenic capacity of bacterial isolates) as a conservation strategy.

## Materials and Methods

### Site Description and Sampling

**FIGURE 1:**
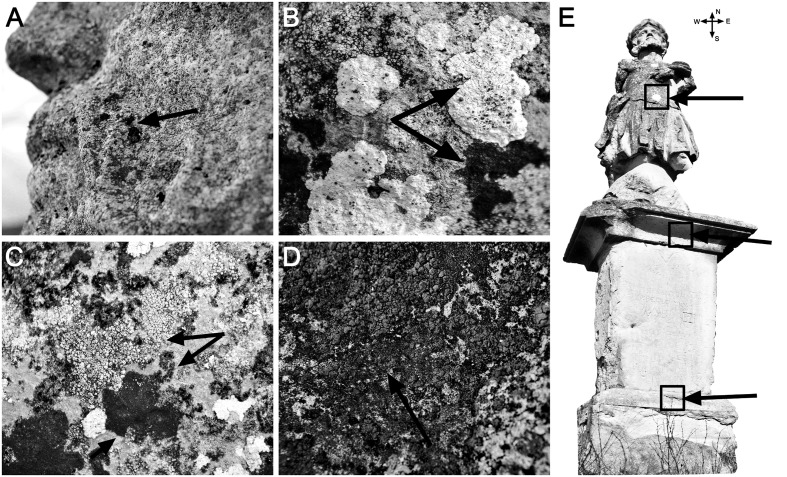
**Close-up images (A–D)** of the Saint Donatus statue showing visible signs of microbial colonization and biodeterioration (indicated by black arrows), and a statue image **(E)** showing the collection points for the “southern” samples (indicated by black arrows).

The baroque statue of Saint Donatus (46°46′28.77′′ N; 23°33′26.49′′ E) is located in the NE part of Cluj-Napoca city (Cluj County), on a hill near the Hoia forest. This limestone monument was built in 1740 and is considered to be the city’s first public statue (Sabãu, 2002). Due to its centuries-long exposure to the outdoor environment, at the time of sampling (July 2013) the stone monument presented visible signs of esthetic and structural damage caused by microorganisms (**Figure 1**). The epilithic samples were collected from 16 cm^2^ squares using sterile scalpels (without causing any visible damage to statue), placed into sterile tubes and transported on ice to the laboratory, where they were immediately processed. The samples were taken from three different zones along the axis of each cardinal point (i.e., north, south, east, and west), and from the upper and lower parts of the monument (in agreement with the four cardinal directions).

### Community-Level Physiological Profiling

The metabolic profiles of the epilithic bacterial communities inhabiting Saint Donatus statue were assessed using BIOLOG EcoPlates (BIOLOG Inc., Hayward, CA, USA). Each 96-well plate is divided into three parts, each one containing 31 carbon sources and one water blank as negative control. During microbial respiration the tetrazolium redox dye is reduced, leading to a purple coloration, which is further measured at 590 nm. For each sample, 130 μl of the bacterial suspension (containing approximately 10^6^ cells diluted in 0.25x Ringer’s solution) was used to inoculate the plates. After inoculation, the plates were incubated in the dark under aerobic conditions for 120 h. The optical density (OD_590_) was measured after each 24 h and up to 5 days, using the FLUOstar Omega microplate reader (BMG Labtech, Cary, NC, USA). From the initial reads obtained we subtracted the absorbance of the negative control (the minimum absorbance considered as positive was 0.30). The data was further analyzed using the software GraphPad Prism 6 (San Diego, CA, USA).

### Fingerprinting Analysis of the Bacterial Communities

The whole-community DNA was extracted from the collected samples by using the ZR Soil Microbe DNA MiniPrep kit (Zymo Research, Irvine, CA, USA) according to the protocol provided by the manufacturer. The 16S rRNA genes were amplified using the primer pair 27F/1492R (Supplementary Table S1) on a Palm Cycler^TM^ (Corbett Research, Mortlake, NSW, Australia) machine. The PCR reaction mixture contained the following components: 0.5 μM of each 27F and 1492R primers, 0.2 μM of each deoxynucleoside triphosphates (dNTPs), 1x buffer, 20 mM of MgCl_2_, 5 μL template DNA, 1.5 U of TaqPol and RNase/DNase-free water to a final volume of 20 μL. The reactions were carried out as following: 180 s initial denaturation at 95°C, followed by 35 cycles of: 30 s denaturation at 95°C, 30 s primer annealing at 56°C and extension for 90 s at 72°C. The obtained PCR products were further used as template in a nested PCR, in order to obtain approximately 200 bp fragments of the 16S rRNA genes. The primer pair used for the nested PCR was 341-GC/518R (Supplementary Table S1). All PCR reactions were performed in triplicate in 50 μL volumes containing: 0.5 μM of the forward (341-GC) and reverse (518R) primers, 0.2 μM of each dNTPs, 1x buffer, 20 mM of MgCl_2_, 1 μL template DNA, 1.5 U of TaqPol and RNase/DNase-free water to a final volume of 50 μL. The reactions were carried out as following: 120 s initial denaturation at 94°C, followed by 22 cycles of: 30 s denaturation at 94°C, 30 s primer annealing at 58°C and extension for 30 s extension at 72°C.

For genetic fingerprinting of the bacterial communities present in our samples, 150 μL of nested PCR products were purified using GeneJET PCR Purification Kit (Thermo Scientific, Waltham, MA, USA) following the protocol provided by the manufacturer, and then separated by denaturing gradient-gel electrophoresis (DGGE) on 10% polyacrylamide gel containing a gradient of 30–55% of denaturants (100% of denaturant containing 40% w/v formamide and 7 M urea). The gradient was prepared using the gradient former model 485 (Bio-Rad, Hercules, CA, USA) and a peristaltic pump. The gel was run in 1% TAE using the DCode^TM^ Universal Mutation Detection System (Bio-Rad, Hercules, CA, USA) for 16 h at 50 V and 61°C. After electrophoresis, the gel was stained for 25–30 min in a solution of 1% TAE containing 1 × SYBR Gold^®^ (Invitrogen, Carlsbad, CA, USA) and then visualized and photographed in a UVP Imaging and Analysis System (Upland, CA, USA). The bacterial community DGGE fingerprints were analyzed with the BioNumerics 6.5 software package (Applied Maths, Ghent, Belgium) according to the instructions provided by the developer.

### Bacterial Estimation by Quantitative Real-Time PCR (qPCR)

Quantitative real-time PCR was performed to estimate the relative abundance of bacteria on Saint Donatus statue. The bacterial amplification reactions were carried out in triplicate, using iCycler IQ5 Real-Time System (Bio-Rad, Hercules, CA, USA) with the EvaGreen DNA-binding dye. The reaction mixture contained the following components: 7 μL 1 × SsoFast EvaGreen SuperMix (Bio-Rad, Hercules, CA, USA), 0.4 μM of the forward (338F) and reverse (518R) primers (Supplementary Table S1), 10 ng of DNA and RNase/DNase-free water to a final volume of 14 μL. The reactions were carried out as follows: 180 s initial denaturation at 98°C, followed by 45 cycles of: 25 s denaturation at 98°C, 25 s primers annealing at 61°C, and 30 s extension at 72°C. For assessing the specificity of the primers, a post-PCR melting curve analysis was performed, in which the temperature varied between 60 and 90°C in 0.5°C increments with subsequent plate readings. Reaction efficiencies and data analyses were assessed using the background subtracted data and LinRegPCR software (Ruijter et al., 2009).

### Bacterial Cultivation and Phenotypic Characterization

Small amounts from each sample were aseptically suspended in 10 mL of sterile 0.25x Ringer’s solution. From the obtained microbial suspensions 10^-5^ and 10^-6^ serial dilutions were cultured on Nutrient Agar (Oxoid^TM^, Thermo Scientific, Waltham, MA, USA) in sterile Petri dishes at 25°C for 2–3 days. Subsequently, the isolated bacterial colonies (a total of 104) were Gram stained, tested for KOH lysis (Buck, 1982), observed by light microscopy, and assessed for their capacity to precipitate CaCO_3_ by transferring them into Petri dishes containing M-3 solid media [1% Bacto Casitone, 1% Ca(CH_3_COO)_2_ × 4H_2_O, 0.2% K_2_CO_3_ × 1/2H_2_O, in distilled water, pH 8] (Rodriguez-Navarro et al., 2003), and incubating them for 4–5 days at 28°C.

### Phylogenetic Identification of the Isolated Bacterial Strains

Genomic DNA from the isolated bacterial strains (a total of 104 strains) was extracted using GeneJet Genomic DNA Purification Kit (Fermentas-Thermo Scientific, Waltham, MA, USA) following the manufacturer’s instructions and stored at -20°C until further use. The bacterial 16S rRNA genes from the isolated strains were amplified using the primer pair 27F/1492R (Supplementary Table S1) and the same PCR protocol as described above. The obtained PCR products were purified using GeneJET PCR Purification Kit (Thermo Scientific, Waltham, MA, USA) following the protocol provided by the manufacturer and sequenced by Sanger’s method at a commercial company (Macrogen Europe, The Netherlands). The sequences obtained were checked for quality using myRDP Pipeline (Cole et al., 2009), and all sequences that had a minimum length of 600 bp with a *Q*-score > 20 were downloaded and further used. To determine the approximate phylogenetic affiliations, sequences were compared using both GenBank BLASTN and RDP Classifier search tools^1^ (Cole et al., 2014). The graphical representation of the taxonomic tree was constructed with GraPhlAn software (Asnicar et al., 2015).

### Fourier Transform Infrared Spectroscopy (FTIR), X-Ray Diffraction (XRD), and Scanning Electron Microscopy (SEM) Analysis of Biogenic Carbonates

From a total of 104 strains, only 22 strains were able to grow on M-3 agar media and precipitate minerals. The minerals formed by these bacterial strains were collected from the solid media using sterile scalpels, transferred into sterile 2 mL Eppendorf tubes, then purified using the method described by Ferrer et al. (1988). After the isolation and purification procedures, the crystals were analyzed by FTIR. The spectra were recorded on a conventional 4100 Jasco FTIR spectrometer using an ATR sampling device (ZnSe crystal, one reflection). The spectral resolution was 4 cm^-1^ and the number of scans was 32. Further XRD analysis of the crystals formed by the 22 bacterial strains were carried out on a Bruker D8 Advance diffractometer with CoKα1 and λ = 1.78897, Fe filter and a one-dimensional detector, using corundum (NIST SRM1976a) as internal standard. The data were collected on a 5–64° 2θ interval, at a 0.02° 2θ, with the measuring step of 0.2 s. The identification of mineral phases was performed with the Diffrac.Eva 2.1 software from Bruker AXS, using the PDF2 (2012) database. The morphology of the CaCO_3_ deposits formed by the bacterial isolates on M-3 agar media was visualized by SEM. After fixation with 100% hexamethyldisilazane (Electron Microscopy Sciences, Hatfield, PA, USA) and ethanol dehydration, samples were air-dried and then coated with a 10 nm gold layer, and visualized with a FEI Quanta 3D FEG, and a Hitachi SU8230 scanning electron microscopes, both operated at 30 kV.

### Nucleotide Sequence Accession Numbers

The 16S rRNA gene sequences corresponding to the isolated bacterial strains have been deposited in GenBank under the accession numbers KX842369–KX842447S.

## Results

### Community-Level Physiological Profiling

The community-level physiological profiling showed that the epilithic microbial communities present on the Saint Donatus statue were metabolically active and able to utilize a wide range of carbon sources present in the BIOLOG EcoPlates assay. The results showed that after 5 days of incubation all six tested microbial communities (from the northern, southern, eastern, western, upper, and lower parts of the statue) were able to metabolize 16 out of 31 organic carbon sources (i.e., pyruvic acid, Tween 40 and 80, D-cellobiose, β-methyl-D-glucoside, D-xylose, D-mannitol, n-acetyl-D-glucosamine, D-glucosaminic acid, glucose-1-phosphate, D-galactonic acid γ-lactone, D-galacturonic acid, itaconic acid, D-malic acid, L-asparagine and L-serine). At the end of the incubation period, the microbial community residing in the southern side of the statue utilized the highest number of available substrates (83.9% from the total available organic carbon substrates), while the communities dwelling on the eastern and upper parts utilized the lowest numbers of available carbon sources (61.3 and 64.5%).

The time-substrate usage relation (Supplementary Figure S1) showed that microbial functional diversity changed over time, and that the microbial community present in the lower part of the statue was less metabolically active in comparison with the others. Furthermore, the microbial assemblages proved to be capable of growth after 24 h of incubation and to initially prefer substrates from the carboxylic and amino acids groups (Supplementary Figure S1).

### Spatial Distribution of Bacterial Abundance and Community Fingerprinting by PCR-DGGE

The estimation of bacterial numbers on the Saint Donatus statue performed by qPCR indicated that the highest average abundance (cells/cm^2^) was found in the upper part (1.62 × 10^8^), where the microbial biofilms were more abundant and visible. Cell densities of approximately 10^7^ were detected in all the other samples, while the lowest bacterial density (1.62 × 10^7^ cells/cm^2^) was found in the lower part of the statue (Supplementary Figure S2).

**FIGURE 2:**
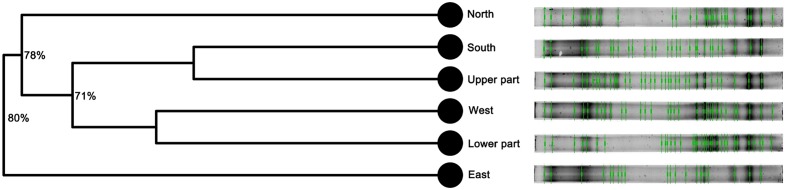
**Fingerprinting profiles based on PCR-DGGE and cluster analysis (UPGMA) of the bacterial communities inhabiting Saint Donatus statue performed suing Bionumerics 6.5 software**. The branch quality of the cluster analysis is shown as the Cophenetic correlation coefficient.

The bacterial fingerprinting analysis performed by PCR-DGGE showed that all the tested samples have distinct microbial community structures (**Figure 2**). The number of bands present in each sample, together with the Margalef species richness index (Supplementary Table S2) indicated that the most phylogenetically diverse community is present in the upper part of the statue. The fingerprinting profiles also indicated that the bacterial assemblages dwelling in the southern and upper part, as well as the ones from the western and lower part of the statue grouped together.

### Cultivation, Identification, and Carbonatogenetic Potential of the Isolated Bacterial Strains

**FIGURE 3:**
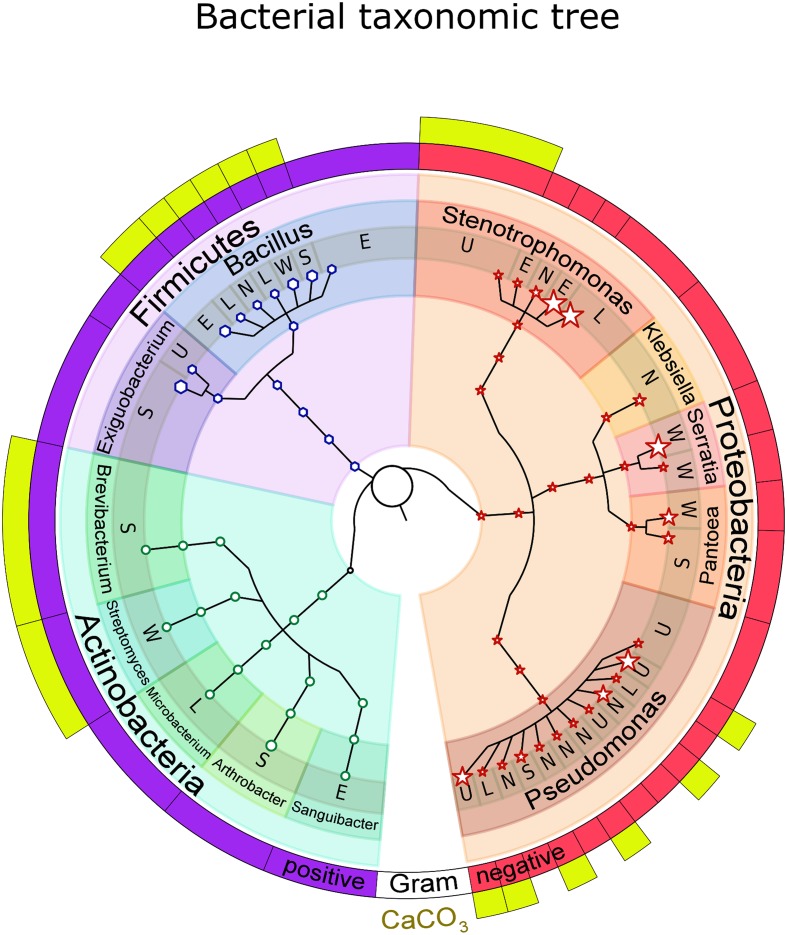
**Taxonomic tree of the bacterial strains isolated from Saint Donatus statue**. The node sizes reflect the microorganisms’ abundance. The annotations above the terminal nodes of the tree indicate the isolation location on the statue: N (North), S (South), E (East), W (West), U (Upper part), and L (Lower part). The first external circle shows the Gram coloration results, while the second one (with yellow boxes) highlights the bacteria capable of CaCO_3_ precipitation. The number within the first clade marker represents the total number of isolated bacterial strains that received taxonomic identification.

From the cultivated bacterial strains we selected 15–20 strains/sample (104 in total), which we characterized molecularly and phenotypically. After DNA isolation, 16S rRNA gene amplification and sequencing, we obtained 79 high-quality sequences that were further utilized in taxonomic identification (Supplementary Table S3). From the 79 bacterial isolates 73.4% were found to belong to Proteobacteria, while Firmicutes and Actinobacteria counted for 19 and 7.6%, respectively (**Figure 3**). The majority of bacteria were found to be Gram-negative (73.4%) and belonged to the *Pseudomonas* (32.9%) and *Stenotrophomonas* (21.5%) genera. Strains from the *Serratia*, *Pantoea*, *Bacillus*, *Exiguobacterium*, *Arthrobacter*, and *Brevibacterium* genera were also found to be common among the isolated *Bacteria* (**Figure 3**). While the isolates from the northern, eastern, western, upper and lower parts of the statue were mostly Gram-negative, the isolates from the southern part proved to be predominantly Gram-positive (Supplementary Table S3).

**FIGURE 4:**
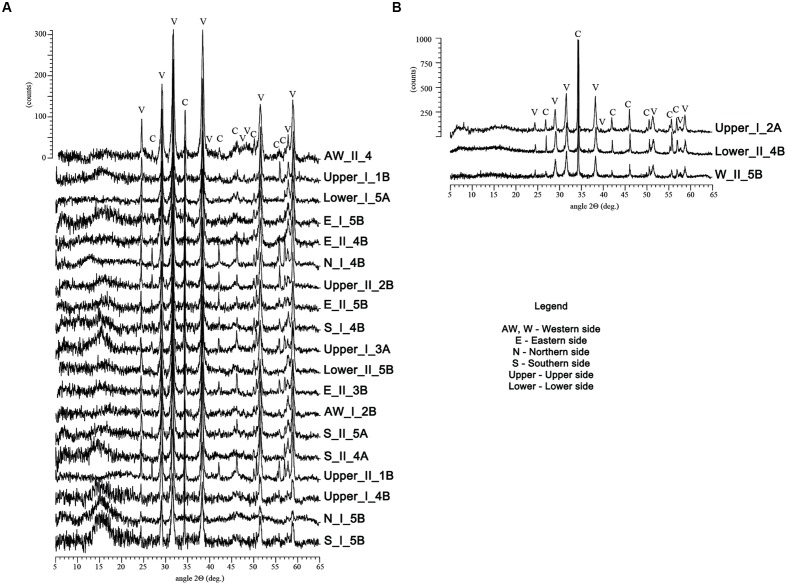
**X-ray diffraction patterns of the crystals recovered from the M-3 agar media recorded on a Bruker D8 Advance diffractometer**. The patterns of the samples in the **(A)** image were predominated by vaterite (V), while the ones from the **(B)** contained predominantly calcite (C). The *y*-scale (intensity) was fairly similar for each group of samples.

**FIGURE 5:**
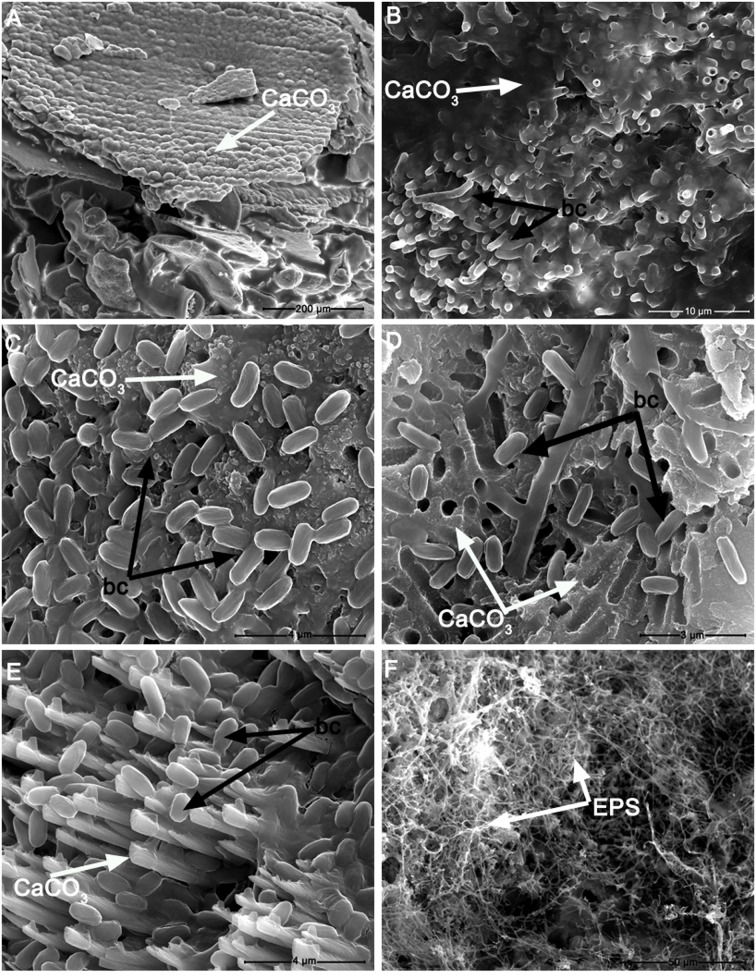
**Scanning electron microscopy photomicrographs of: (A)** calcium carbonate precipitate (CaCO_3_) induced by *Stenotrophomonas chelatiphaga* on M-3 agar medium; **(B)** detail of calcified *S. chelatiphaga* cells (bc) forming tubular vaterite structures growing from the calcium carbonate matrix (CaCO_3)_; **(C)** detail of *Bacillus licheniformis* cells (bc) attached to a calcium carbonate matrix (CaCO_3_); **(D)** transversal section of a calcium carbonate crust showing *B. pumilus* cells and a porous calcium carbonate matrix (CaCO_3_) which presents cavities left by the embedment of bacterial cells during carbonatogenesis; **(E)** *B. pumilus* cells and vaterite crystals (CaCO_3_); **(F)** Exopolysaccharide (EPS) film formed by *Streptomyces albidoflavus* grown on M-3 agar medium.

**FIGURE 6:**
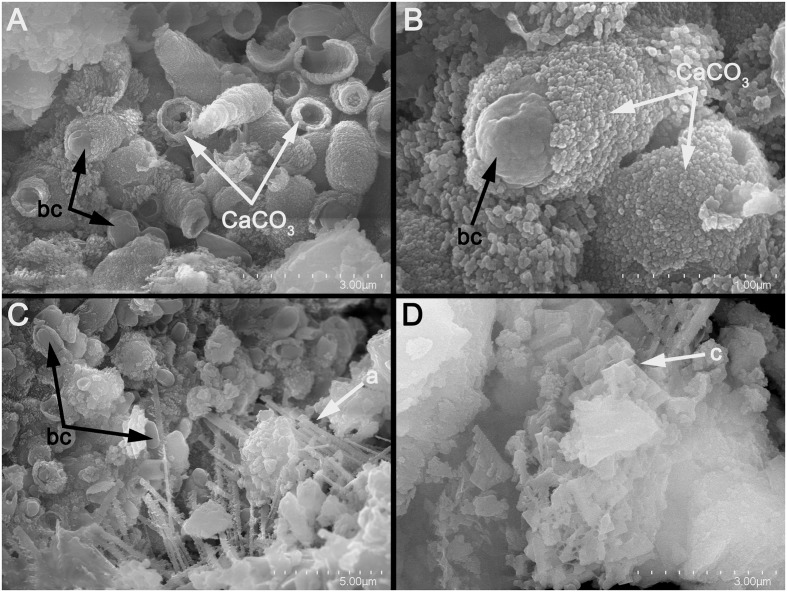
**Scanning electron micrographs of: (A)** tubular vaterite constructions induced by *B. pumilus*; **(B)** detail of **(A)**, showing the distribution of vaterite aggregates on the bacterial cells; **(C)** *B. pumilus* and aragonite needle-like crystals (a); **(D)** Calcite crystals (c) induced by *B. pumilus*.

Out of the 79 bacterial strains that were further grown on M-3 agar media, 22 were found to be capable of CaCO_3_ precipitation. These isolates were classified within the *Bacillus* (50% of total carbonatogenic strains), *Pseudomonas* (36.4% of total carbonatogenic strains), *Brevibacterium*, *Streptomyces*, and *Stenotrophomonas* (each 4.5% of total carbonatogenic strains) genera (Supplementary Table S3). The CaCO_3_ crystals were further isolated, purified and analyzed by FTIR, XRD, and SEM. The FTIR spectra (Supplementary Figure S3) showed the presence of absorption bands at 712 and 742 cm^-1^ in all the crystals produced by the 22 bacterial strains, suggesting the presence of vaterite and calcite. The XRD patterns indicated that in most samples vaterite (the hexagonal CaCO_3_ polymorph) was the dominant phase, while calcite, the rhombohedral CaCO_3_ polymorph, was present in a lesser extent. Aragonite, the orthorhombic calcium carbonate, was tentatively ascribed at 30.6 2θ (in sample Awest_I_2B) being near the estimated 3% detection limit for X-ray powder diffraction. The intensity scale in **Figure 4** shows a relatively poor crystallinity for all the phases, whereas the background “hump” in the 12–18 2θ interval may account for amorphous calcium carbonate still present in the samples. The SEM analyses were performed in order to confirm the FTIR and XRD data, and to determine the calcium carbonate crystal morphology. The SEM images (**Figures 5**, **6**) suggest that amorphous calcium carbonates coexist with crystalline polymorphs. Furthermore, the analysis of microbial colonies revealed mineralized bacterial cells (**Figures 5B**, **6B**) that form porous micrometer-sized calcium carbonate sheets (**Figures 5A,D**). The SEM imaging also indicated that the carbonate crystals were formed by the interaction of bacteria with their growth media and that they acted as nucleation sites for CaCO_3_ precipitation, leading to their entrapment and formation of calcium carbonate alveoles (**Figures 5**, **6**). The alveoles consisted of nanoscale vaterite aggregates, whose hexagonal symmetry is quite difficult to ascertain (**Figure 6**); however, some calcite rhombohedrons and needle-like aragonite crystals have also been found (**Figure 6**).

## Discussion

Biomineralization is a widespread phenomenon of minerals precipitation mediated by microorganisms. One common natural occurring biomineralization process is the microbial carbonate precipitation, which has been vastly studied and described in different environments (such as caves, soils, sediments, aquifers and open-water areas) as playing an important role in the cementation of these ecosystems (Boquet et al., 1973; Cañveras et al., 2001; Banks et al., 2010; Chou et al., 2011). Over the past few years, microbial carbonate precipitation has emerged as a promising method for conservation and restoration of limestone artwork by offering multiple advantages in comparison with traditional procedures (Le Métayer-Levrel et al., 1999; Dhami et al., 2014b). Although it has been shown that limestone could be consolidated by applications of culture media inoculated with the desirable bacterial strains (i.e., with high carbonatogenetic ability and that produce a precipitate similar to the substrate) such as *Myxococcus xanthus* (Rodriguez-Navarro et al., 2003) and some *Pseudomonas* and *Bacillus* species (Cameotra and Dakal, 2012), a more advantageous method relies on using the resident microbiota that has the ability to induce calcium carbonate precipitation (Ettenauer et al., 2011; Jroundi et al., 2012). Since the efficiency of the last method is purely dependent on the microbial communities already present on the stonework, we investigated if single or multiple bacterial assemblages with biomineralization potential could colonize a cultural heritage monument (the Saint Donatus statue).

The molecular fingerprinting analysis performed by PCR-DGGE displayed complex, community-specific band patterns. Additionally, it indicated that the bacterial assemblages, in spite of their high phylogenetic diversity, were dominated by a few abundant ribotypes (corresponding to the high-intensity bands). As the surrounding soil is often considered the source of stonework microbiota (Walsh, 2001) and its DGGE profiles are generally characterized by high species evenness (Smalla et al., 2007), the bacterial communities inhabiting the Saint Donatus statue appeared to be site-specific. Although the UPGMA analysis revealed that the band patterns of each sample clustered separately, it also showed that the bacterial communities were characterized by the presence of common ribotypes.

Since microbial carbonate precipitation is influenced by the metabolic activity of the bacterial communities (Seifan et al., 2016; Zhu and Dittrich, 2016), we investigated their metabolic potential by using BIOLOG Ecoplates assays. As ammonification is an important metabolic pathway involved in CaCO_3_ precipitation (Zhu and Dittrich, 2016), the BIOLOG EcoPlates experiments highlighted that all the investigated microbial communities have the capacity to use amino acids as carbon and nitrogen sources (indicating their potential utilization in bioconsolidation). As metabolization of amino acids favors alkalinization (caused by the ammonia released in the process of oxidative deamination), it creates the pH conditions necessary for biomineralization (Jimenez-Lopez et al., 2008). Moreover, by knowing that the microbial communities present on the statue have the possibility to perform ammonification (of amino acids) we selected a culture media (i.e., M-3) for the biomineralization experiments, which contained bacto-casitone as nitrogen and carbon source. By choosing this substrate we intended to obtain bacterial mediated carbonate precipitation via bicarbonate formation and CaCO_3_ supersaturation (González-Muñoz et al., 2010).

The community-level physiological profiling showed that the heterotrophic bacterial communities from the eastern and lower part of the statue had a reduced metabolic activity by comparison with the rest (as inferred from OD measurements). The diminished metabolic activity of the bacterial communities dwelling on the east side of the statue corroborates with a lower phylogenetic diversity as showed by the PCR-DGGE analysis. On the other hand, the reduced metabolic capacity of the bacterial communities inhabiting the lower part of the statue corresponds to a high phylogenetic diversity (as shown by molecular fingerprinting). We consider that this result may be explained by the proximity of the sampling site to the ground, which facilitates a higher inoculation of the statue by microbial spores and soil microbiota that are not yet metabolically adapted to the ecological niches of the statue. Furthermore, Braun et al. (2006) showed that growth of soil bacteria is not supported by α-cyclodextrin, D,L-α-glycerol phosphate and α-ketobutyric acid, substrates that were not used by the community inhabiting the lower part of the statue (by comparison α-ketobutyric acid and α-cyclodextrin were used by the southern communities).

The number of epilithic bacteria present on the Saint Donatus statue was high (average of 6.425 × 10^7^ cells/cm^2^) and comparable with the one reported for Maya limestone artworks (McNamara et al., 2006), indicating that biomineralization could be used as a preservation method. The numbers of bacteria were found to be in the same order of magnitude for all the tested communities, with the exception of the upper part of the statue, which was covered by a visible green biofilm and had a higher bacterial abundance. We think that this higher bacterial density could be explained by the fact that heterotrophic bacterial communities thrive in the proximity of phototrophs (Warscheid and Braams, 2000).

The cultivation procedures allowed the isolation of both Gram-negative and -positive bacteria, with the former greatly outnumbering the latter. This finding is in agreement with the study performed by Kiel and Gaylarde (2006), which reports similar results for the epilithic microbiota colonizing historical buildings. This higher number of culturable Gram-negatives might also be explained by the repeated inoculation of the statue with microbiota originating from the surroundings, as many bacteria isolated from biodeteriorated stone artworks were proved to be typical for soils (Warscheid and Braams, 2000; Walsh, 2001). The predominance of Gram-positive bacteria on the southern part of the statue may be attributed to their higher capacity to resist intense sunlight and desiccation for longer periods of time (Warscheid and Braams, 2000). The culture-based approach revealed that the predominant isolated Bacteria phylum was Proteobacteria followed by Firmicutes and Actinobacteria, in agreement with other studies performed on stone artwork (Ortega-Morales et al., 2004; Suihko et al., 2007; Lan et al., 2010).

From the isolated bacterial strains, 22 proved to be capable to grow on M-3 media and precipitate calcium carbonate. Amongst them, the genera *Bacillus* (represented by *B*. *pumilus*, *B*. *licheniformis*, *B*. *safensis*, and *B*. *altitudinis*) and *Pseudomonas* (represented by *P*. *putinda*, *P*. *graminis*, *P*. *fluorescens*, *P*. *synxantha*, and *P*. *oryzihabitans*) were found to be the most prevalent. *B*. *licheniformis* was previously detected on stone monuments (McNamara et al., 2006) and cave systems (Baskar et al., 2008), whereas *B*. *pumilus* was found to be present in soils, sediments, caves and seawater (Boquet et al., 1973; Baskar et al., 2006; Silva-Castro et al., 2013; Daskalakis et al., 2015). Furthermore, all the five *Pseudomonas* species were previously shown to be widely spread in nature, being found to inhabit stone monuments, caves, rivers and soils (Boquet et al., 1973; Laiz et al., 1999; Shirakawa et al., 2011; Rusznyák et al., 2012). To the authors’ knowledge this is the first study that shows the biomineralization capacity of: *Pseudomonas synxantha*, *P. graminis*, *Brevibacterium iodinum*, *Streptomyces albidoflavus*, and *Stenotrophomonas chelatiphaga*.

Although the M-3 media contained calcium acetate, no precipitates were found in the sterile control experiments, indicating that a purely biotic process was responsible for carbonate formation. The SEM analyses showed that the most prevalent CaCO_3_ polymorph (i.e., vaterite) consisted of nanoscale aggregates formed directly onto the surface of bacterial cells. This observation is in agreement with recent findings that pinpoint to the formation of vaterite nanoparticles as a common process in microbial biomineralization (Rodriguez-Navarro et al., 2007; Jroundi et al., 2014). Furthermore, the SEM observations of fossilized bacterial cells were reported in other studies performed on biogenic calcium carbonate formation (Rodriguez-Navarro et al., 2003, 2007; Daskalakis et al., 2015). We considered that the affinity of vaterite nanoparticles to bacterial cell walls may be induced by electrostatic and stereochemical interactions at the inorganic-organic interface and by media supersaturation in the manner described by Rodriguez-Navarro et al. (2003).

Although bacteria have the capacity to induce the formation of different calcium carbonate polymorphs (vaterite, aragonite, and calcite), the reasons underlying their selection are not well understood and believed to be linked to metabolic activity, cell wall characteristics, extracellular polymeric substances and growth media composition (Seifan et al., 2016). Being a metastable polymorph of CaCO_3_, vaterite is scarcely found in natural environments, where it is generally associated with bacterial carbonatogenesis (Rodriguez-Navarro et al., 2003). Noteworthy was the fact that under our experimental conditions most of the microbial strains induced vaterite formation, a polymorph with a high solubility that may be prone to dissolution by meteoric waters and to a low degree of consolidation (by comparison to calcite). However, recent findings suggest that the microbially-induced vaterite achieves similar degrees of stability as calcite by incorporating organic molecules (Jimenez-Lopez et al., 2008; Jroundi et al., 2014).

We consider that in the biomineralization experiments the calcium carbonate precipitation was influenced by the cell surfaces, which acted as effective nucleation templates (see SEM images). Due to their capacity to adsorb ions (Mg^2+^, Fe^3+^, Cu^2+^, Na^+^, K^+^, Mn^2+^, Zn^2+^, Ca^2^, etc.) the bacterial cell walls most likely increased the Ca^2+^ concentration in the microenvironment, creating the prerequisite for CaCO_3_ precipitation (Dhami et al., 2014a; Zhu and Dittrich, 2016). What is more, we consider that the extracellular polymeric substances (**Figure 5F**) contributed in a similar way to carbonates precipitation, by binding divalent cations (Tourney and Ngwenya, 2014). In our opinion, the observed biomineralization occurred as a cumulative effect of the nucleation sites (i.e., bacterial cell walls) and the metabolic activity of the cells. Due to the fact that the bacterial oxidative deamination of amino acids (present in the M-3 culture media) leads to bicarbonate formation (Zhu and Dittrich, 2016) and that Ca^2+^ are concentrated in the cell-wall proximity, the ion activity product meets the requirement to exceed the solubility constant and trigger precipitation in the way described by Dhami et al. (2014a).

Furthermore, we reason that the formation of vaterite on the cell walls and in their proximity is in agreement with Ostwald’s rule of stages (Mann, 2001), by which stable phase formation may be preceded by metastable phases (e.g., vaterite), which are favored under non-equilibrium conditions (increased calcium and carbonate concentration, high pH). The fact that the precipitated vaterite was stable after 1 year (data not shown) indicated that solid-state transition did not occurred, suggesting its stabilization by organic compounds and its biogenic origin (Daskalakis et al., 2015). The above-mentioned arguments according to which calcium carbonate precipitation is a consequence of bacterial metabolic capacity (i.e., ammonification of amino acids) and cell wall structure are corroborated by the taxonomy results (by which calcium carbonate precipitation was induced by different bacterial genotypes), and are in agreement with recent findings that suggest that biomineralization is a widespread phenomenon (Zhu and Dittrich, 2016).

In general, biomineralization leads to the production of different phases of anhydrous CaCO_3_ polymorphs such as calcite, vaterite and aragonite, but hydrated phases of CaCO_3_ have also been reported (Wei et al., 2003; Ben Chekroun et al., 2004; Gebauer et al., 2010; Dhami et al., 2013). Among these polymorphs the most common precipitated forms are calcite and vaterite (Rodriguez-Navarro et al., 2007; Rusznyák et al., 2012; Dhami et al., 2014a), the former being the most dominant and thermodynamically stable polymorph of microbially-precipitated calcium carbonate (Stocks-Fischer et al., 1999; Okwadha and Li, 2010). Through FTIR and XRD analysis, we have showed that the carbonatogenetic isolated strains produce mainly vaterite and calcite.

Fourier transform infrared spectroscopy has already proven its potential to differentiate between the three crystalline forms of calcium carbonate phases: calcite, aragonite, vaterite (Sato and Matsuda, 1969; Vagenas et al., 2003). In spite of the overlapping of several bands, Vagenas et al. (2003) proposed marker absorption bands which were found to be suitable for the quantitative analysis of calcium carbonate phases in ternary mixtures: 713 cm^-1^ for calcite, 700 and 713 cm^-1^ for aragonite, and 745 cm^-1^ for vaterite. Since the 700 cm^-1^ band does not appear in the analyzed samples, the two main crystalline forms observed were found to be vaterite and calcite. Although the samples generally have low crystallinity, the XRD patterns are well resolved, accounting mostly for vaterite and calcite, with aragonite being present in one sample. Although the majority of bacteria that we tested for their capacity to induce CaCO_3_ precipitation produced vaterite, we consider that a restoration method based on biomineralization may be feasible, since this biogenic induced calcium carbonate polymorph was shown to confer similar degrees of consolidation as calcite (Jimenez-Lopez et al., 2008; Jroundi et al., 2014).

## Conclusion

The employed methodology indicated that the epilithic bacterial communities inhabiting Saint Donatus statue are different in relation to their spatial localization, although they share some common species. Despite the fact that the bacterial assemblages proved to be dissimilar, they were all found to contain strains able to induce mineralization by calcium carbonate precipitation. Furthermore, we expanded the current knowledge on culturable diversity of carbonatogenic bacteria by identifying five additional bacterial species. Based on the results of this study, the authors emphasize the need to evaluate the carbonatogenetic potential of all the bacterial communities present on stonework prior to designing an efficient conservation treatment based on biomineralization.

## Author Contributions

A-ŞA, MP, and HLB designed the research and wrote the manuscript. A-ŞA, MP, TT, NH, NL, and LB-T performed the experiments and analyzed the data. All the authors discussed the results and commented on the manuscript.

## Conflict of Interest Statement

The authors declare that the research was conducted in the absence of any commercial or financial relationships that could be construed as a potential conflict of interest.

## Footnotes

1 blast.ncbi.nlm.nih.gov
