# Supplementary material for: Diversity and Biomineralization Potential of the Epilithic Bacterial Communities Inhabiting the Oldest Public Stone Monument of Cluj-Napoca (Transylvania, Romania)
Source: Front Microbiol. 2017 Mar 7;8:372. doi: 10.3389/fmicb.2017.00372 (PMC5339310; doi:10.3389/fmicb.2017.00372)
Supplement: Supplementary file 1 [file Data_Sheet_1.pdf]

Supplementary Material

Diversity and Biomineralization Potential of the Epilithic Bacterial Communities Inhabiting the Oldest Public Stone Monument of Cluj-Napoca (Transylvania, Romania)

Adrian-Ştefan Andrei[1,2,~], Manuela Raluca Păuşan[1,3,~], Tudor Tămaș[4], Nicolae Har[4], Lucian Barbu-Tudoran[1,5], Nicolae Leopold[6] and Horia Leonard Banciu[1,7*]

[1]Department of Molecular Biology and Biotechnology, Babeş-Bolyai University, Cluj-Napoca, Romania
[2]Institute of Hydrobiology, Department of Aquatic Microbial Ecology, Biology Center of the Academy of Sciences of the Czech Republic, České Budějovice, Czech Republic
[3]Department for Internal Medicine, Medical University of Graz, Graz, Austria
[4]Department of Geology, Babeş-Bolyai University, Cluj-Napoca, Romania
[5]Electron Microscopy Center, Babeş-Bolyai University, Cluj-Napoca, Romania
[6]Faculty of Physics, Babeş-Bolyai University, Cluj-Napoca, Romania
[7]Center for Systems Biology, Biodiversity, and Bioresources, Babes-Bolyai University, Cluj-Napoca, Romania.

[~]These authors have equal contribution to this work.

[*]**Correspondence**: Horia Leonard Banciu, Faculty of Biology and Geology, Babes-Bolyai University, 5-7 Clinicilor Str., 400006 Cluj-Napoca, Romania. Tel.: +40-264-431691; fax: +40-264-431858; e-mail: Horia.Banciu@ubbcluj.ro

Supplementary tables

Supplementary Table S1. Primers used in PCR amplification.

Primer name	Target Group	Primer sequence (5' to 3')	Reference
**1492R**	Universal	GGTTACCTTGTTACGACTT	(Lane, 1991)
**27F**	Bacteria	AGAGTTTGATCCTGGCTCAG	(Lane, 1991)
**338F**	Bacteria	ACTCCTACGGGAGGCAGCAG	(Lane, 1991)
**518R**	Bacteria	ATTACCGCGGCTGCTGG	(Muyzer et al., 1993)
**341-GC**	Bacteria	ACGGGGGGCCTACGGGAGGCAGCAG	(Muyzer et al., 1993)

Supplementary Table S2. The number of distinct PCR-DGGE bands and the Margalef index for the analysed microbial communities. The samples names reflect their location on Saint Donatus statue.

Samples	Number of bands	Margalef species richness
North	28	3.989
South	31	4.539
East	20	2.973
West	31	4.317
Upper	34	4.813
Lower	32	4.488

Supplementary Table S3. 16S rRNA gene-based taxonomy of the bacterial strains isolated from the Saint Donatus statue and their Gram coloration. The table contains the closest GenBank sequence match, together with its accession number and sequence identity.

Sample	BLASTN-based taxonomy	Identity	Gram staining
North_I_1A	*Stenotrophomonas rhizophila* (KR055001.1)	99%	Negative
North_I_2A	*Pseudomonas synxantha* (KC834326.1)	100%	Negative
North_I_4A	*Pseudomonas synxantha* (KC834326.1)	100%	Negative
North_II_1A	*Pseudomonas grimontii* (KR054989.1)	100%	Negative
North_II_2A	*Pseudomonas synxantha* (NR_113583.1)	100%	Negative
North_II_3A	*Stenotrophomonas rhizophila* (KR055001.1)	100%	Negative
North_II_4A	*Pseudomonas synxantha* (KC834326.1)	99%	Negative
North_I_3B	*Klebsiella sp*. OS-B4 (FN178363.1)	99%	Negative
North_I_4B*	*Pseudomonas fluorescens* (KU291443.1)	99%	Negative
North_I_5B*	*Bacillus pumilus* (KJ476724.1)	99%	Positive
North_II_1B	*Klebsiella sp*. OS-B4 (FN178363.1)	99%	Negative
North_II_2B	*Pseudomonas lurida* (KM891560.1)	99%	Negative
North_II_4B	*Pseudomonas sp*. SGb394 (HQ224639.1)	99%	Negative
South_I_1A	*Exiguobacterium undae* (FN870071.1)	99%	Positive
South_I_2A	*Arthrobacter luteolus* (DQ486130.1)	100%	Positive
South_I_3A	*Arthrobacter luteolus* (DQ486130.1)	99%	Positive
South_I_4A	*Exiguobacterium undae* (AB669472.1)	99%	Positive
South_I_5A*	*Bacillus pumilus* (KJ476724.1)	99%	Positive
South_II_2A	*Pantoea agglomerans* (AF130947.2)	99%	Negative
South_II_3A	*Exiguobacterium undae* (AB669472.1)	99%	Positive
South_II_4A	*Pantoea agglomerans* (KR265469.1)	99%	Negative
South_II_5A*	*Bacillus pumilus* (KJ476724.1)	100%	Positive
South_I_1B	*Pseudomonas putida* (KF640247.1)	98%	Negative
South_I_4B*	*Pseudomonas putida* (KF640247.1)	99%	Negative
South_I_5B*	*Brevibacterium iodinium* (KM259941.1)	99%	Positive

East_I_1A	*Sanguibacter keddieii* (KF911337.1)	100%	Positive
East_I_2A	*Stenotrophomonas rhizophila* (KR055001.1)	100%	Negative
East_I_4A	*Stenotrophomonas rhizophila* (KR055001.1)	100%	Negative
East_II_2A	*Stenotrophomonas rhizophila* (KR055001.1)	99%	Negative
East II 4 A	*Stenotrophomonas rhizophila* (KR055001.1)	100%	Negative
East_I_3B	*Stenotrophomonas rhizophila* (KR055001.1)	99%	Negative
East_I_5B*	*Bacillus pumilus* (KJ476724.1)	99%	Positive
East_II_1B	*Stenotrophomonas rhizophila* (KR055001.1)	95%	Negative
East_II_2B	*Stenotrophomonas rhizophila* (KR055001.1)	99%	Negative
East_II_3B*	*Bacillus safensis* (KJ672329.1)	99%	Positive
East_II_4B*	*Bacillus pumilus* (KJ476724.1)	99%	Positive
East_II_5B*	*Bacillus licheniformis* (KU363978.1)	99%	Positive
West_I_1B	*Serratia marcescens* (KC790279.2)	100%	Negative
West_I_2B	*Serratia marcescens* (KC790279.2)	100%	Negative
West_I_3B	*Serratia marcescens* (KC790279.2)	99%	Negative
West_I_4B	*Serratia marcescens* (KC790279.2)	100%	Negative
West_II_1B	*Serratia marcescens* (KC790279.2)	100%	Negative
West_II_2B	*Serratia marcescens* (KC790279.2)	100%	Negative
West_II_3B	*Serratia sp*. M401 (KJ944090.1)	100%	Negative
West_II_4B	*Serratia marcescens* (KC790279.2)	100%	Negative
West_II_5B*	*Streptomyces albidoflavus* (KT758342.1)	100%	Positive
AWest_II_1	*Pantoea sp*. CH-N10 (HQ396801.1)	99%	Negative
AWest_II_2	*Pantoea sp*. CH-N10 (HQ396801.1)	99%	Negative
AWest_I_2B*	*Bacillus pumilus* (KJ476724.1)	100%	Positive
AWest_I_3B	*Pantoea sp*. CH-N10 (HQ396801.1)	99%	Negative
AWest_II_3	*Pantoea sp*. CH-N10 (HQ396801.1)	99%	Negative
AWest_II_4*	*Bacillus pumilus* (KJ476724.1)	97%	Positive
Upper_I_1A	*Pseudomonas sp*. UT1 (JX133179.1)	99%	Negative
Upper_I_2A*	*Stenotrophomonas chelatiphaga* (NR_116366.1)	99%	Negative
Upper_I_3A*	*Pseudomonas oryzihabitans* (KJ401059.1)	100%	Negative
Upper_II_1A	*Pseudomonas oryzihabitans* (KJ401059.1)	100%	Negative
Upper_II_2A	*Pseudomonas oryzihabitans* (KJ401059.1)	99%	Negative
Upper_II_3A	*Pseudomonas oryzihabitans* (KJ401059.1)	100%	Negative

Upper_II_4A	*Exiguobacterium sibiricum* (LK391534.1)	99%	Positive
Upper_I_1B*	*Pseudomonas synxantha* (KC834326.1)	100%	Negative
Upper_I_2B	*Pseudomonas putida* (KT767824.1)	98%	Negative
Upper_I_3B	*Pseudomonas synxantha* (KC834326.1)	100%	Negative
Upper_I_4B*	*Pseudomonas synxantha* (NR_113583.1)	99%	Negative
Upper_II_1B*	*Pseudomonas oryzihabitans* (KJ401059.1)	99%	Negative
Upper_II_2B*	*Pseudomonas oryzihabitans* (KJ401059.1)	100%	Negative
Upper_II_3B	*Pseudomonas synxantha* (KC834326.1)	100%	Negative
Upper_II_4B	*Pseudomonas synxantha* (KC834326.1)	99%	Negative
Lower_I_1A	*Stenotrophomonas rhizophila* (KR055001.1)	99%	Negative
Lower_I_2A	*Stenotrophomonas rhizophila* (KR055001.1)	99%	Negative
Lower_I_4A	*Stenotrophomonas rhizophila* (KR055001.1)	99%	Negative
Lower_I_5A*	*Bacillus altitudinis* (KJ826579.1)	99%	Positive
Lower_II_1A	*Stenotrophomonas rhizophila* (KR055001.1)	100%	Negative
Lower_II_2A	*Microbacterium oxydans* (LN890028.1)	100%	Positive
Lower_II_3A	*Stenotrophomonas rhizophila* (KR055001.1)	100%	Negative
Lower_II_4A	*Stenotrophomonas rhizophila* (KR055001.1)	99%	Negative
Lower_I_1B	*Pseudomonas rhizosphaerae* (KF147111.1)	99%	Negative
Lower_II_2B	*Stenotrophomonas rhizophila* (KR055001.1)	99%	Negative
Lower_II_4B*	*Pseudomonas graminis* (KF147075.1)	99%	Negative
Lower_II_5B*	*Bacillus pumilus* (KJ476724.1)	99%	Positive

* Bacterial strains showed to be carbonatogenic

Supplementary figures

Supplementary Figure S1

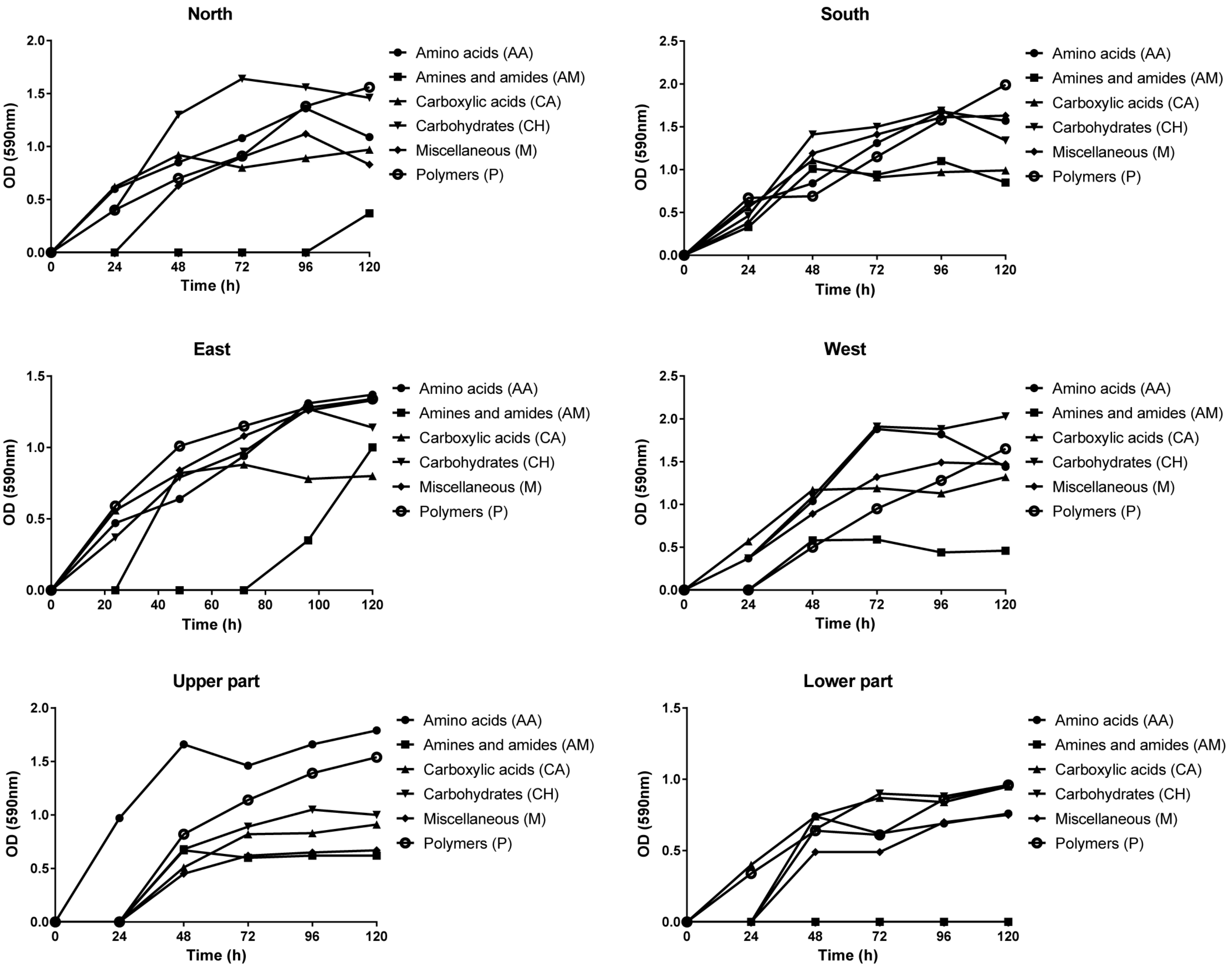

Time variation of the average well colour development in the BIOLOG EcoPates resulted from organic carbon substrate utilization by the microbial communities present on Saint Donatus statue.

Supplementary Figure S2

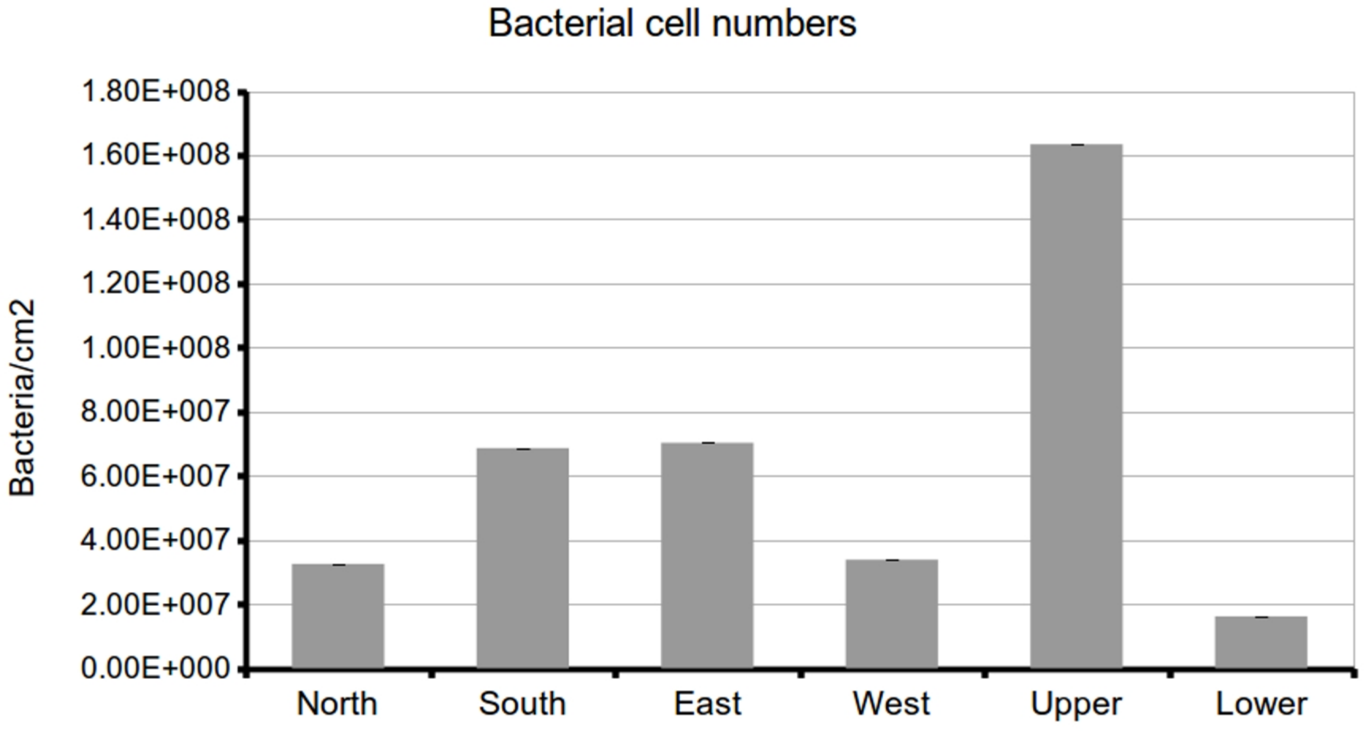

Bacterial cell numbers/cm^2 as estimated by quantitative real-time PCR. The sampling sites are represented on the abscissa. The columns represent means and the error bars (black colour) represent s.d. values.

Supplementary Figure S3

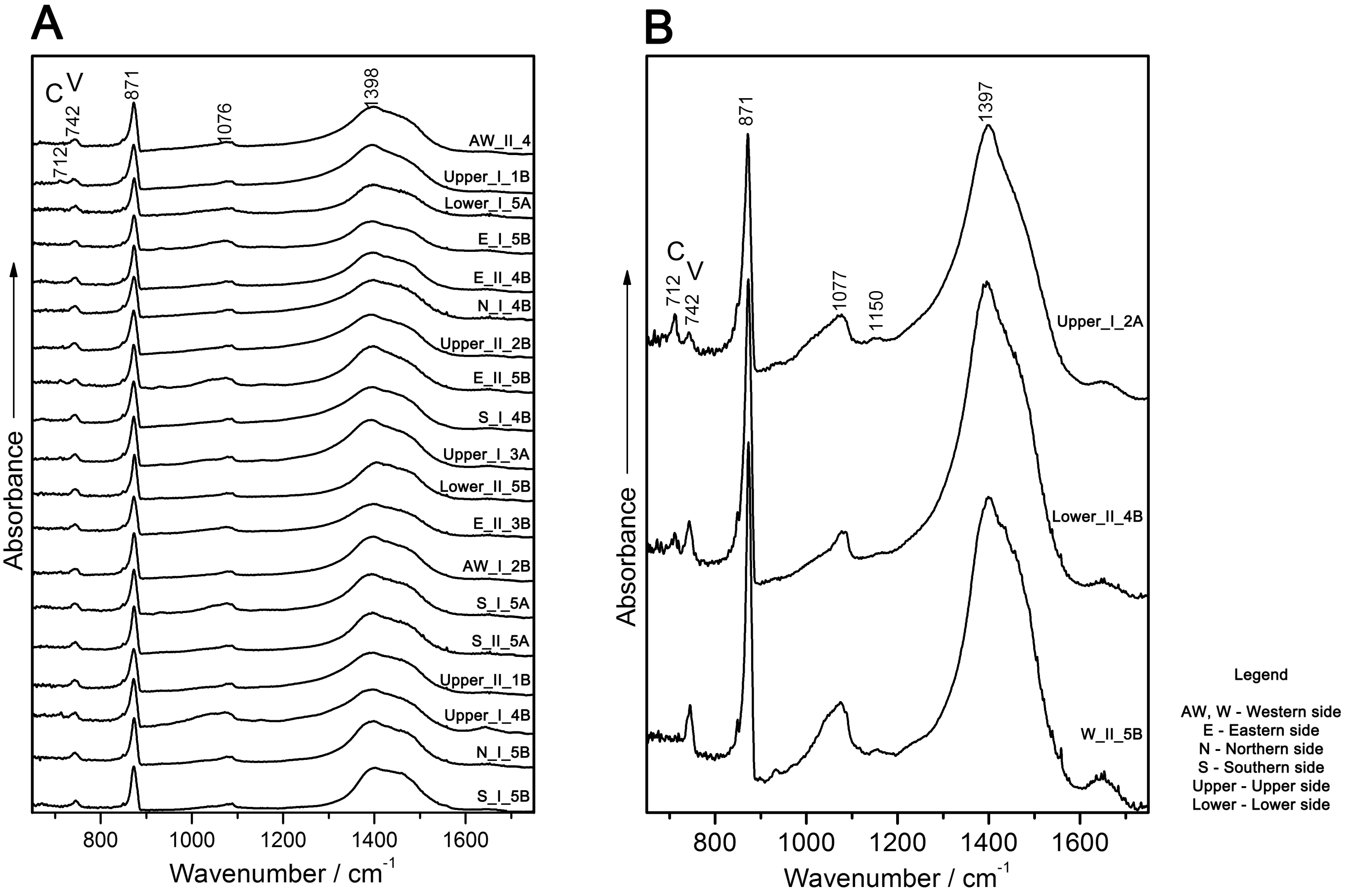

FTIR spectra of the crystals recovered from the M-3 agar media recorded on a conventional 4100 Jasco FTIR spectrometer using an ATR sampling device (ZnSe crystal, one reflection). The spectral resolution was 4 cm^{-1} and the number of scans was 32. The samples in the A image were mostly vaterite (V), while the ones from the B image contained predominantly calcite (C).
